# Natural History of Left Ventricle Thrombus After ST-Segment Elevation Myocardial Infarction: An Echocardiographic Contrast Study

**DOI:** 10.1016/j.jacadv.2023.100329

**Published:** 2023-05-24

**Authors:** Rami M. Abazid, Andrew Frost, Usha Manian, Rodrigo Bagur, Nikolaos Tzemos

Left ventricular thrombus (LVT) is a well-recognized complication after ST-segment elevation myocardial infarction (STEMI),^1^ which if left untreated gives rise to a significant increase in thromboembolic events. Although the incidence of LVT has markedly reduced with primary percutaneous coronary intervention (PPCI), it still remains high at up to 10%.^1^ The use of a vitamin K antagonist for a period of at least 3 to 6 months is considered the anticoagulation regimen of choice for treatment of LVT.^2^ To date, the pattern of regression and resolution of LVT is not well described. We aimed to analyze the natural history of LVT resolution using contrast-enhanced echocardiography during clinical follow-up.

Consecutive patients with LVT early post-STEMI (during hospitalization) were prospectively enrolled between January 2020 and December 2020. All patients underwent PPCI with drug-eluting stents and were treated with triple therapy (warfarin, aspirin, and ticagrelor) for 1 year as well as usual guideline-directed medical therapy (GDMT). International normalized ratio (INR) was closely monitored to maintain a therapeutic level above 2.5 in all patients during follow-up. Individuals with a prior indication for anticoagulation, at high bleeding risk with HAS-BLED score ≥3, and those with previous LVT or preexisting left ventricular ejection fraction (LVEF) <55% % before the indexed hospitalization for STEMI were excluded. All echocardiography studies were contrast-enhanced and performed at baseline/post-PCI and every 3 months for a period of 12 months. After injection of the ultrasound enhancing agent, a zoomed view that demonstrated the largest LVT dimensions was obtained and the focus was adjusted to obtain the best image resolution. A very low mechanical index was used (<0.20), which allowed an optimal left ventricular opacification. These images were analyzed using Xcelera workstations (Philips Healthcare). All studies were evaluated by 2 experienced echocardiographers. Major bleeding was defined as intracerebral hemorrhage, or bleed resulting in hemodynamic compromise that requires treatment. The study was approved by the local ethics committee, and all patients signed informed consent.

All analyses were conducted using SPSS Statistics for Windows, version 28 (IBM Corp). Continuous normally distributed variables were represented as mean ± SD and compared between groups using Student’s *t*-test. Categorical variables were represented as frequencies and percentages and were compared using chi-square test. A multivariable regression model was built by including all variables that were deemed to be significant with univariable regression analysis using a cutoff *P* value of 0.05.

**Figure 1:**
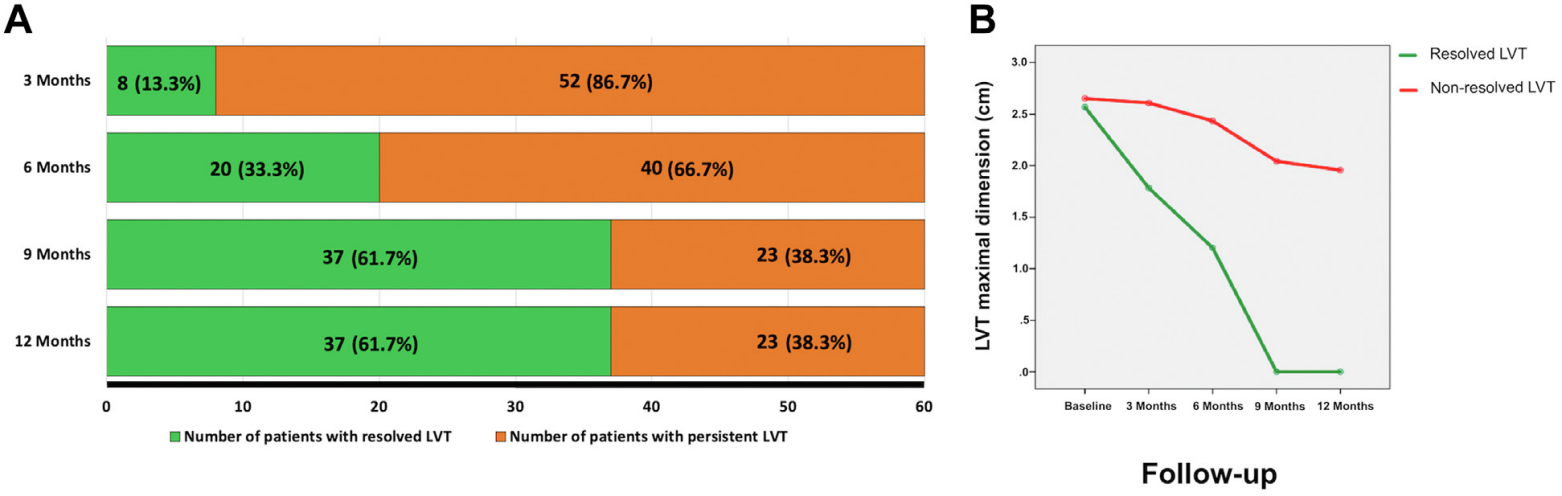
LVT Follow-up **(A)** Left ventricular thrombus resolution over the follow-up period. **(B)** Left ventricular thrombus size regression in each group throughout the follow-up period. LVT = left ventricular thrombus.

Sixty patients were enrolled with a mean age of 64 ± 9 years, 41 (68%) were men. During the follow-up period, the number of patients with LVT was: 60 (100%) at baseline, 52 (86.7%) at 3 months, 40 (66.7%) at 6 months, 23 (38.3%) at 9 months and 23 (38.3%) at 12 months (Figure 1A).

We found that patients with complete LVT resolution had a higher prevalence of dyslipidemia (29/37 [78.4%] vs 9/23 [39.1%], *P* = 0.003), a higher LVEF at baseline (40% ± 4% vs 35.3% ± 4%, *P* < 0.0001) and at 1 year (47.2% ± 6% vs 40.5% ± 5%, *P* < 0.0001), when compared to patients with persistent LVT. Conversely, a history of previous myocardial infarction was less common in patients with resolution of LVT: (9/37 [24.3%] vs 15/23 [65.2%], *P* = 0.002).

Furthermore, Patients with complete LVT resolution showed no significant differences in the prevalence of diabetes mellitus (12/37 [32.4%] vs 4/23 [17.4%], *P* = 0.16), initial LVT dimension (2.6 ± 0.5 cm vs 2.7 ± 0.5 cm, *P* = 0.48), presence of regional dyskinesia (29/37 [78.4%] vs 14/23 [60.8%], *P* = 0.50); of note, other baseline characteristics did not differ significantly.

Univariate regression analysis showed that baseline LVEF, 1-year LVEF and dyslipidemia were significantly associated with LVT resolution. However, multivariable regression analysis including these 3 variables demonstrated that LVEF at 1 year (OR: 0.84; 95% CI: 0.73-0.98; *P* = 0.014) was the only variable associated with LVT resolution. Additionally, thrombus size did not change significantly over the follow-up period among patients with persistent LVT (2.7 ± 0.5 cm vs 1.9 ± 0.5 cm, *P* = 0.84) (Figure 1B). Neither major bleeding nor thromboembolic events were reported during study period.

Little is known about the natural history of LVT in the modern era of invasive PPCI and guideline-directed medical therapy. We performed a prospective study, with stringent anticoagulation control and state-of-the-art echocardiographic assessment to gain insight into the factors for the resolution of an LVT following STEMI. We found that only 61.6% of patients had complete LVT resolution at 9 months with no further change in resolution out to 12 months, despite therapeutic anticoagulation (INR 2.5-3). Importantly, LVEF at 12 months was the only predictor for complete LVT resolution. This could be explained by the fact that a lower LVEF is associated with increased LV remodeling and wall motion abnormalities which may decrease the likelihood of LVT resolution. In this study, no major bleeding events were reported which could be explained by the small sample size, stringent anticoagulation control (INR target: 2.5-3.0), monthly INR checks at anticoagulation clinic, exclusion of patients with high bleeding risk (comparatively low HAS-BLED score), and a relatively young population with a mean age of 64 years.

We limited the prospective follow up to 12 months since the persistence or absence of LVT beyond this time may not impact the choice of discontinuing anticoagulation. In conclusion, approximately 62% of patients with LVT post-STEMI had complete LVT resolution at 9 months and 12 months on triple therapy (warfarin, aspirin, and ticagrelor).
